# Relationship of glycemic variability with delirium and mortality among critically ill elderly patients with sepsis: A retrospective matched cohort study

**DOI:** 10.1371/journal.pone.0339707

**Published:** 2026-05-18

**Authors:** Jianqin Huang, Jiexuan Xu, Yu Zhai, Shuting Liu, Pengcheng Duan, Jiayan Liang, Yile Tian, Murong Lu, Xuemei Liu, Hongjing Yu

**Affiliations:** 1 Intensive Care Unit, The Second Affiliated Hospital Of Guangzhou Medical University, Guangzhou, Guangdong, China; 2 Department Of Cardiovascular Medicine, The Second Affiliated Hospital of Guangzhou Medical University, Guangzhou, Guangdong, China; 3 Nursing Administration Department, The Second Affiliated Hospital of Guangzhou Medical University, Guangzhou, Guangdong, China; University of Sao Paulo: Universidade de Sao Paulo, BRAZIL

## Abstract

**Objective:**

The associations of glycemic variability (GV) with the occurrence of delirium and 90-day and 180-day mortality in critically ill patients with sepsis remain unclear. This study aimed to investigate the associations of glycemic variability with the development of delirium and death.

**Method:**

This study employed a retrospective analysis of elderly sepsis patients admitted to the intensive care unit (ICU) for the first time. Patients were categorized into two groups based on their GV: a high-risk group (< 21.399%) and a low-risk group (≥21.39%). The relationship between GV and delirium was assessed via logistic regression and restricted cubic splines. Cox regression was employed to analyze the relationship between GV and patients’ 90-day and 180-day mortality.

**Results:**

This study included 12228 elderly patients who were diagnosed with sepsis. The high-risk group presented a significantly elevated risk of delirium (27.9% vs 22.1%) and higher 90-day (40.0% vs 30.1%) and 180-day (46.0% vs 35.7%) mortality rates (all p < 0.001). The increase in GV was approximately nonlinear with increasing risk of delirium. In addition, high GV was associated with the greatest risk among delirium (OR = 1.122, 95% CI 1.023–1.230), 90-day mortality (HR = 1.149, 95% CI 1.07–1.227) and 180-day mortality (HR = 1.153, 95% CI 1.085–1.224) in elderly sepsis patients.

**Conclusion:**

GV is independently associated increased risk of delirium and 90-day and 180-day mortality in elderly sepsis patients, indicating that GV can serve as a biomarker for identifying at higher risk of delirium and mortality in sepsis patients.

## 1. Introduction

Sepsis represents a life-threatening condition characterized by a dysregulated host response to infection, commonly progressing to multiple organ dysfunction and elevated fatality rates [1]. Beyond its impact on short-term survival, sepsis frequently results in long-term physical and psychological illness, significantly increasing the risk of hospital readmission after discharge [2,3]. Sepsis-associated delirium (SAD) is a common and severe neurological complication characterized by acute brain dysfunction. It presents with altered mental status, impaired attention, cognitive decline, and perceptual disturbances [4]. In elderly patients, delirium frequently arises from intricate interactions among multiple factors. Within the intensive care unit (ICU), this condition is associated with a range of adverse outcomes, including prolonged hospitalization, increased mortality, and reduced quality of life [5,6]. Given its clinical burden, early identification of patients at high risk for delirium is critical. Identifying potential biomarkers associated with SAD is crucial for improving prognosis.

Contemporary research has established glycemic variability (GV) as a significant metric, offering enhanced insight into the pathophysiological consequences of disrupted metabolic homeostasis compared to static glucose measurements. Stress hyperglycemia occurs in nearly 24% of patients admitted to intensive care units and is associated with an increased risk of complications [7,8]. These findings highlight the pivotal role of glucose homeostasis in sepsis patients. Importantly, marked fluctuations in blood glucose appear to exert even more detrimental effects [9]. The guideline advocates the need for research to identify safe and effective strategies for achieving optimal glycemic control, reducing hypoglycemia, and refining glucose management across different patient populations [1]. GV serves as a metric developed by researchers to quantify fluctuations in blood glucose [10]. Current evidence demonstrates that elevated GV is associated with an increased risk of cardiovascular diseases, endothelial injury, insulin resistance, and inflammation [11–13]. Recent studies suggest that substantial glycemic fluctuations may contribute to brain injury through mechanisms such as oxidative stress, mitochondrial dysfunction, and disruption of the blood–brain barrier [14,15]. However, validation of these hypotheses in sepsis remains preliminary. Clinical evidence has centered on the associations between GV and postoperative delirium in individuals undergoing cardiac surgery, demonstrating that higher GV levels are associated with a greater risk of delirium [16,17]. Additionally, elevated GV has been linked to adverse outcomes in various acute conditions, including heart failure, acute pancreatitis, and traumatic brain injury [18–20]. While research on GV in sepsis remains limited, existing evidence indicates that high GV on the first day of sepsis is associated with early adverse outcomes [21]. Moreover, the impact of high GV on mortality appears to be more pronounced in patients with more severe sepsis [22]. These findings indicate that effective glycemic control may play an important role in mitigating the risk of poor outcomes.

Despite this, current evidence regarding the influence of glycemic variability on delirium and outcomes in elderly patients with sepsis remains limited. Given the potential value of GV, this retrospective study aimed to examine the correlation between GV and the incidence of delirium, as well as 90-day and 180-day mortality in this patient population.

## 2. Materials and methods

### 2.1. Study design, setting and population

This retrospective observational cohort study employed the Medical Information Mart for Intensive Care-IV (MIMIC-IV-3.1), which is a publicly available and comprehensive database [23]. The database includes anonymized data from 2008-- 2022, sourced from the intensive care units of the Beth Israel Deaconess Medical Center in Boston [24,25]. It comprises detailed demographics, laboratory data, nursing notes, diagnostic records, medication logs, and other critical health information.

**Fig 1:**
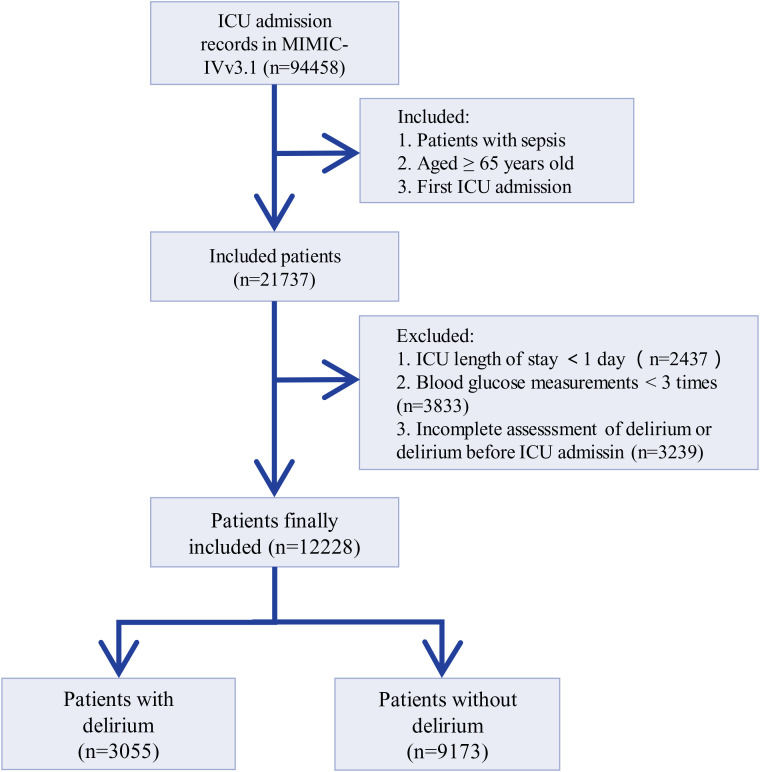
Flowchart of patients’ inclusion and exclusion.

This study included only patients who were aged ≥ 65 years, diagnosed according to the Sepsis-3 criteria [26], and were initially admitted to the ICU. We excluded the following patients: (1) whose ICU stay was less than one day; (2) whose blood glucose measurements were less than three times; and (3) who had experienced delirium before ICU admission or were not assessed for delirium during ICU stay (Fig 1). This study followed the STROBE (Strengthening the Reporting of Observational Studies in Epidemiology) guidelines for reporting [27].

### 2.2. Data collection

Our researchers utilized Navicat Premium software (version 15.0.12) and structured query language (SQL) to extract data from the MIMIC-IV database. The extracted data included demographic information, vital signs, laboratory indicators, therapy, comorbidities, and disease severity scores. To reduce potential bias, variables exhibiting missing data exceeding 30% were excluded from analysis. Vital signs, laboratory indicators, disease severity scores, and therapy data were extracted from the first measurement within 24 hours after ICU admission. Comorbidities were identified primarily using ICD-9 and ICD-10 codes. The missing data rate did not exceed 30%. For variables with less than 30% missing data, multiple imputations were conducted using the ‘MICE’ package in R software, employing the random forest method.

We computed the Time-Weighted Average Glucose (TWAG) for all study participants by integrating every biochemical glucose measurement obtained during their intensive care unit admission. To address variability in blood glucose sampling intervals, TWAG was utilized as an alternative indicator of mean glycemic levels, enabling individualized patient assessment. The Gi denotes the i-th blood glucose measurement, and ΔTi represents the time interval between the i-th and (i-1)-th measurements. The formula for the TWAG is as follow: [28]


$$TWAG\left(\frac{mg}{dL}\right)=\frac{\sum \left(\left(G_{i}+G_{i-\text{1}}\right)\left(\frac{mg}{dL}\right)\times \Delta T_{i}(h)\right)}{\text{2}\sum \Delta T_{i}(h)}$$

Additionally, the coefficient of variation (CV) is a blood glucose variability-related indicator used to quantify GV, defined as the ratio of the standard deviation (SD) divided by the TWAG and then times 100% [29]. Traditionally, a GV value≤36% has defined the safety threshold [30]. No established standards, however, exist for normal or abnormal GV ranges in critically ill elderly patients with sepsis [31]. Drawing from prior studies of GV in patients with sepsis and heart failure [21,32], we calculated the optimal GV cutoff value of 21.39% that can effectively distinguish the survival risk of patients with 90-day mortality as the outcome (S1 Fig in S1 File). Simultaneously, this is the median value of CV. Based on this, we divided GV into low-risk and high-risk groups.

### 2.3. Outcomes

The main outcome measure in this study was delirium, which was evaluated by medical staff using the Confusion Assessment Method for the Intensive Care Unit (CAM-ICU) [33]. The CAM-ICU evaluates four critical components: (1) acute mental status fluctuations; (2) inattention; (3) altered level of consciousness; and (4) disordered cognition. Delirium is confirmed when concurrent symptoms of both the first and second features, supplemented by either the third or fourth features. For the secondary outcome, we analyzed mortality during 90 and 180 days of follow-up.

### 2.4. Covariates extraction

The selection of covariates for this study was based on a comprehensive approach that included a review of previous studies, clinical practice experience and expertise, and data availability. Other independent variables analyzed for adjustment included age, gender, race, disease severity scores (Acute Physiology Score III (APS III), Simplified Acute Physiology Score II (SAPS II), Charlson Comorbidity Index (CCI), Glasgow Coma Scale (GCS), Sequential Organ Failure Assessment (SOFA)), laboratory indicators (albumin, blood urea nitrogen, lactate, hemoglobin, platelets, white blood cells), comorbidities (myocardial infarction, congestive heart failure, atrial fibrillation, hypertension, cerebrovascular disease, dementia, diabetes, liver disease, cancer) and treatment (vasoactive agent, ventilation, continuous renal replacement therapy (CRRT), benzodiazepines injection, the use of corticosteroid and insulin). Due to multicollinearity, the Model 3 was not included in SAPS II and CCI.

### 2.5. Statistical analysis

Patients were stratified into two cohorts based on GV: a low-risk group (GV < 21.39%) and a high-risk group (GV ≥ 21.39%). The normality of continuous variables was assessed via the Shapiro-Wilk test. Normally distributed data are presented as mean ± standard deviation, whereas non-normally distributed data are expressed as median with interquartile range (IQR). Categorical variables were presented as numbers and percentages. The Wilcoxon rank-sum test and the chi-square test were used to assess group differences. Univariate and multivariate logistic regression were used to assess the association between CV and the risk of delirium. Prior to multivariate logistic regression, the variance inflation factors (VIFs) were used to check for multicollinearity between variables. Restricted cubic splines (RCSs) were plotted to illustrate the association between CV and delirium. Cox proportional hazard regression was used to examine the associations between CV and 90-day mortality and 180-day mortality. Results were reported as hazard ratios (HRs) or odds ratios (ORs) with 95% confidence intervals (95% CIs). By plotting the Kaplan–Meier (K–M) survival curves, the relationship between the CV and the survival probability of elderly patients with sepsis in the ICU was investigated. The log-rank test was used to compare the curves between the high-risk groups and the low-risk groups. Statistical analyses were performed using R version 4.4.1. Subgroup analyses were conducted according to the prespecified subgroups including sex, race, myocardial infarction, atrial fibrillation, cerebrovascular disease, dementia, diabetes, vasoactive agents and ventilation.

### 2.6. Sensitivity analysis

To evaluate the robustness of our findings, propensity score matching (PSM) was used to mitigate potential estimation bias by adjusting for confounding factors in observational datasets. Analyses were adjusted for factors including sex, race, APS III, GCS, albumin, blood urea nitrogen, lactate, and myocardial infarction, congestive heart failure, atrial fibrillation, hypertension, cerebrovascular disease, dementia, diabetes, liver disease, cancer and vasoactive agent, ventilation, CRRT, benzodiazepines, corticosteroid, insulin, enteral nutrition, parenteral nutrition, GCS, APS III, SAPS II, SOFA, CCI. Optimal matching employing logistic regression calculated patient propensity scores through a 1:1 matching protocol. Following matching, standardized mean differences (SMDs) were computed to assess intergroup balance regarding baseline characteristics.

### 2.7. Ethics statement

Given that MIMIC-IV is a publicly available, de-identified dataset that typically does not require additional institutional ethical approval for secondary analysis. After completing the necessary training and assessments, our researchers have secured a licence (record ID: 13578377) to use the database.

## 3. Results

### 3.1. Sociodemographic and clinical characteristics

This study included 12228 elderly patients who were diagnosed with sepsis. Their baseline characteristics are detailed in Table 1. The median age of the cohort was 76.86 years (IQR: 70.83–83.76 years), with 5,583 patients (45.7%) being female and 8209 (67.1%) being white. Patients classified as high-risk (GV ≥ 21.39%) exhibited prolonged hospitalizations, extended ICU admissions, greater disease severity, and increased comorbidity frequency relative to their low-risk counterparts (GV < 21.39%). These high-risk patients also presented a significantly greater risk of adverse outcomes, including delirium and mortality (all *p* < 0.001).

**Table 1:** Baseline characteristics of elderly patients with sepsis in the ICU.

Variability	Overall	Low-risk group: GV < 21.39%*	High-risk group: GV ≥ 21.39%*	P
	(n = 12228)	(n = 6114)	(n = 6114)	
**General characteristics**				
Age, years	76.86 (70.83, 83.76)	77.39 (71.12, 84.21)	76.35 (70.62, 83.25)	<0.001
Rac				<0.001
White	8209 (67.1)	4307 (70.4)	3902 (63.8)	
Black	1276 (10.4)	512 (8.4)	764 (12.5)	
Asian	373 (3.1)	158 (2.6)	215 (3.5)	
Hispanic	333 (2.7)	116 (1.9)	217 (3.5)	
Other	2037 (16.7)	1021 (16.7)	1016 (16.6)	
Sex				<0.001
Male	6645 (54.3)	3432 (56.1)	3213 (52.6)	
Female	5583 (45.7)	2682 (43.9)	2901 (47.4)	
ICU LOS, days	4.11 (2.55, 7.76)	3.77 (2.40, 6.75)	4.62 (2.74, 8.86)	<0.001
Hospital LOS, days	10.70 (6.73, 17.86)	9.83 (6.30, 16.59)	11.65 (7.10, 19.71)	<0.001
**Vital signs**				
Heart rate, times/min	83.41 (73.65, 95.67)	82.61 (73.27, 94.66)	84.19 (74.08, 96.61)	<0.001
Respiratory rate, times/min	19.44 (17.19, 22.33)	19.24 (17.09, 22.04)	19.69 (17.31, 22.57)	<0.001
Spo2, %	97.08 (95.59, 98.43)	96.97 (95.54, 98.34)	97.16 (95.64, 98.50)	<0.001
SBP, mmHg	112.93 (104.60, 124.12)	113.43 (104.91, 125.24)	112.35 (104.38, 123.19)	<0.001
DBP, mmHg	58.90 (53.12, 65.50)	59.25 (53.45, 65.90)	58.47 (52.77, 65.05)	<0.001
Temperature	36.82 (36.60, 37.10)	36.84 (36.63, 37.12)	36.80 (36.58, 37.08)	<0.001
**Laboratory parameters**				
BUN, mg/dL	29.00 (19.00, 48.00)	26.00 (18.00, 43.00)	33.00 (21.00, 52.00)	<0.001
hemoglobin, m/uL	9.40 (8.00, 11.00)	9.50 (8.00, 11.20)	9.30 (7.90, 10.90)	<0.001
Platelets, K/uL	204.00 (148.00, 278.00)	200.00 (147.00, 269.00)	208.00 (150.00, 287.00)	<0.001
WBC, K/uL	13.70 (9.80, 18.90)	13.30 (9.70, 18.20)	14.10 (10.00, 19.40)	<0.001
Lactate, mmol/L	1.70 (1.20, 2.70)	1.60 (1.10, 2.40)	1.90 (1.30, 3.00)	<0.001
Albumin, g/dL	3.10 (2.60, 3.50)	3.10 (2.70, 3.60)	3.00 (2.60, 3.40)	<0.001
**Medication or treatment**				
Vasoactive agent	6268 (51.3)	2829 (46.3)	3439 (56.2)	<0.001
Ventilation	7422 (60.7)	3444 (56.3)	3978 (65.1)	<0.001
Benzodiazepines	2640 (21.6)	1081 (17.7)	1559 (25.5)	<0.001
Enteral Nutrition	3987 (32.6)	1714 (28.0)	2273 (37.2)	<0.001
Parenteral Nutrition	394 (3.2)	139 (2.3)	255 (4.2)	<0.001
CRRT	951 (7.8)	284 (4.6)	667 (10.9)	<0.001
Corticosteroid	11890 (97.2)	5925 (96.9)	5965 (97.6)	0.031
Insulin	9759 (79.8)	4472 (73.1)	5287 (86.5)	<0.001
**Disease severity scores**				
GCS	15.00 (14.00, 15.00)	15.00 (14.00, 15.00)	15.00 (14.00, 15.00)	0.739
SAPS II	43.00 (36.00, 52.00)	42.00 (35.00, 50.00)	45.00 (38.00, 55.00)	<0.001
APS III	50.00 (39.00, 64.00)	46.00 (36.00, 59.00)	54.00 (43.00, 68.00)	<0.001
Charlson Comorbidity Index	6.89 (2.58)	6.58 (2.54)	7.20 (2.57)	<0.001
SOFA	3.00 (2.00, 4.00)	3.00 (2.00, 4.00)	3.00 (2.00, 5.00)	<0.001
**Comorbidities or symptoms**				
Myocardial infarction	2902 (23.7)	1294 (21.2)	1608 (26.3)	<0.001
Congestive heart failure	5549 (45.4)	2566 (42.0)	2983 (48.8)	<0.001
Peripheral vascular disease	1923 (15.7)	886 (14.5)	1037 (17.0)	<0.001
Cerebrovascular disease	2292 (18.7)	1235 (20.2)	1057 (17.3)	<0.001
Dementia	1097 (9.0)	525 (8.6)	572 (9.4)	0.145
Chronic pulmonary disease	3863 (31.6)	1831 (29.9)	2032 (33.2)	<0.001
Paraplegia	802 (6.6)	471 (7.7)	331 (5.4)	<0.001
Renal disease	4273 (34.9)	1828 (29.9)	2445 (40.0)	<0.001
Atrial fibrillation	5724 (46.8)	2885 (47.2)	2839 (46.4)	0.415
Cancer	2143 (17.5)	1090 (17.8)	1053 (17.2)	0.392
Hypertension	4823 (39.4)	2632 (43.0)	2191 (35.8)	<0.001
Diabetes	4692 (38.4)	1630 (26.7)	3062 (50.1)	<0.001
Liver disease	1312 (10.7)	575 (9.4)	737 (12.1)	<0.001
**Outcome**				
Delirium	3055 (25.0)	1350 (22.1)	1705 (27.9)	<0.001
90-day mortality	4291 (35.1)	1843 (30.1)	2448 (40.0)	<0.001
180-day mortality	4994 (40.8)	2180 (35.7)	2814 (46.0)	<0.001

Data are presented as mean (SE) or frequencies (percentage).

Abbreviation: ICU LOS icu length of stay, Hospital LOS hospital length of stay, SBP systolic blood pressure, DBP diastolic blood pressure, SpO_2_ blood oxygen saturation, BUN blood urea nitrogen, WBC white blood cell, CRRT Continuous Renal Replacement Therapy, GCS Glasgow Coma Score, SAPS II Simplified Acute Physiology Score, APS III Acute Physiology Score III, SOFA Sequential Organ Failure Assessment

*The optimal cutoff value of GV was calculated to be 21.39% to divide the population into low-risk and high-risk groups

### 3.2. GV and the risk of delirium

**Fig 2:**
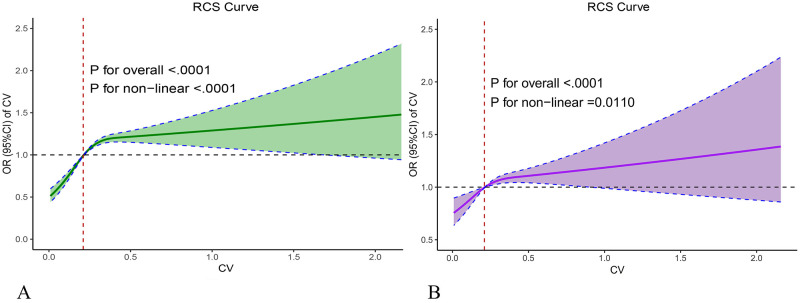
Restricted cubic spline curve of the GV with delirium incidence. **A** for model 1 (unadjusted), **B** for model 3 (adjust for gender, race, bun, lactate, albumin, myocardial infarction, congestive heart failure, atrial fibrillation, hypertension, cerebrovascular disease, dementia, diabetes, liver disease, cancer, vasoactive agent, ventilation, CRRT, benzodiazepines, corticosteroid, insulin, GCS, APS III, SOFA). Abbreviation: GV glycemic variability, CV coefficient of variation, CRRT continuous renal replacement therapy, GCS Glasgow Coma Scale, APS III Acute Physiology Score III, SOFA Sequential Organ Failure Assessment.

Among elderly sepsis patients, delirium prevalence reached 25.0% overall. A significantly higher rate was observed in high-risk individuals (27.9%) compared to their low-risk counterparts (22.1%, *p* < 0.001). We developed three multivariate logistic regression models to investigate the association between GV and delirium incidence. (Table 2). As a continuous variable, each 1% increase in GV was associated with a 1.390-fold increased likelihood of delirium occurrence (95% CI: 1.120–1.734). The results show that delirium risk was significantly amplified in the high-risk patients (OR: 1.122, 95% CI: 1.023–1.230). Furthermore, the restricted cubic spline curves in Fig 2 visually illustrate the linear relationship between CV and delirium risk. Before adjustment, the CV exhibited a nonlinear relationship with delirium (*p* < 0.001), whereas after adjustment, it also showed a nonlinear relationship with delirium (*p* = 0.0226), indicating that the risk of delirium increased with rising CV values above 0.23.

**Table 2:** The association between GV and the risk of delirium among elderly patients with sepsis.

	Model 1		Model 2		Model 3	
	OR (95%CI)	P	OR (95%CI)	P	OR (95%CI)	P
Continuous						
CV, %	1.996 (1.622-2.461)	<0.001	2.016 (1.639-2.492)	<0.001	1.390 (1.120-1.734)	<0.01
Categorical						
Low-risk	Reference		Reference		Reference	
High-risk	1.365 (1.257-1.482)	<0.001	1.367 (1.259-1.486)	<0.001	1.122 (1.023-1.230)	0.014

We quantified GV using the coefficient of variation (CV, %)

Model 1: unadjusted

Model 2: adjust for gender, race

Model 3: adjust for gender, race, bun, lactate, albumin, myocardial infarction, congestive heart failure, atrial fibrillation, hypertension, cerebrovascular disease, dementia, diabetes, liver disease, cancer, vasoactive agent, ventilation, CRRT, benzodiazepines, corticosteroid, insulin, GCS, APS III, SOFA

Abbreviation: GV glycemic variability, OR odds ratio, CI confidence interval, CRRT continuous renal replacement therapy, GCS Glasgow Coma Scale, APS III Acute Physiology Score III, SOFA Sequential Organ Failure Assessment

### 3.3. GV and mortality

**Fig 3:**
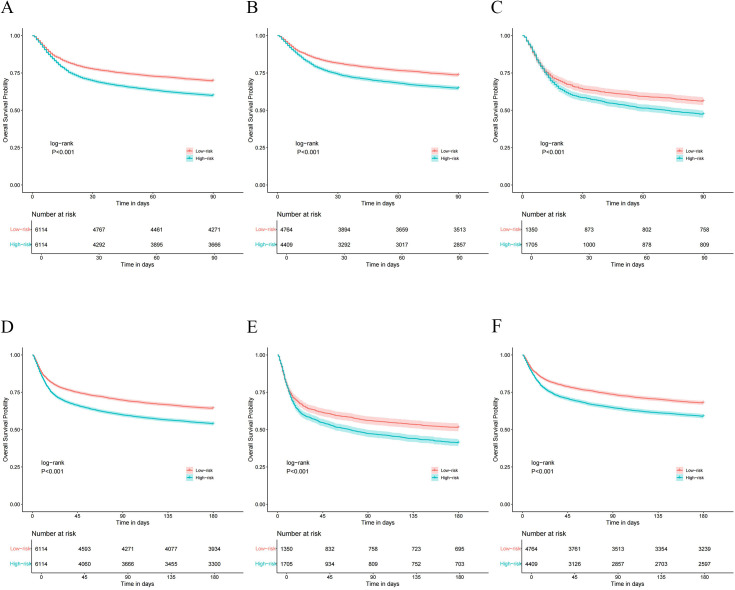
Kaplan–Meier survival analysis curves for 90-day and 180-day mortality. A, B and C for 90-day mortality rate; D, E and F for 180-day mortality rate; A and D for elderly sepsis patients; B and E for elderly sepsis patients with delirium; C and F for elderly sepsis patients without delirium.

We subsequently examined the associations between GV and 90-day and 180-day mortality among the patients. The high-risk cohort exhibited a markedly elevated 90-day mortality rate compared to low-risk patients (40.0% vs 30.1%, *p* < 0.001). Similarly, the high-risk cohort exhibited a markedly elevated 180-day mortality rate compared to low-risk patients (46.0% vs 35.7%, *p* < 0.001) (Table 3). CV was independently associated with increased 90-day mortality (HR 1.296, 95% CI: 1.146–1.465) and 180-day mortality (HR 1.317, 95% CI: 1.174–1.477). Cox regression analysis revealed hazard ratios of 1.149 (95% CI: 1.077–1.227) for 90-day mortality and 1.153 (95% CI: 1.085–1.224) for 180-day mortality in the high-risk group. Furthermore, the application of Kaplan–Meier survival analysis integrated with log-rank test demonstrated statistically significant disparities in mortality at 90-day and 180-day across different GV groups (*p* < 0.001) (Fig 3). Additionally, within both delirious and non-delirious cohorts, significant differences in mortality rates at 90-day and 180-day were observed (*p* < 0.001).

**Table 3:** Cox regression analysis of 90-day and 180-day mortality in the study population among elderly patients with sepsis.

Outcomes	Model 1		Model 2		Model 3	
	HR (95% CI)	P	HR (95% CI)	P	HR (95% CI)	P
90-day mortality						
CV, %	1.483 (1.367-1.608)	<0.001	1.497 (1.381-1.622)	<0.001	1.296 (1.146-1.465)	<0.001
Low-risk	Reference		Reference		Reference	
High-risk	1.415 (1.332-1.504)	<0.001	1.422 (1.339-1.512)	<0.001	1.149 (1.077-1.227)	<0.001
180-day mortality						
CV, %	1.499 (1.388-1.619)	<0.001	1.506 (1.394-1.626)	<0.001	1.317 (1.174-1.477)	<0.001
Low-risk	Reference		Reference		Reference	
High-risk	1.395 (1.319-1.475)	<0.001	1.397 (1.320-1.477)	<0.001	1.153 (1.085-1.224)	<0.001

We quantified GV using the coefficient of variation (CV, %)

Model 1: unadjusted

Model 2: adjust for gender, race

Model 3: adjust for gender, race, bun, lactate, albumin, myocardial infarction, congestive heart failure, atrial fibrillation, hypertension, cerebrovascular disease, dementia, diabetes, liver disease, cancer, vasoactive agent, ventilation, CRRT, benzodiazepines, corticosteroid, insulin, GCS, APS III, SOFA

Abbreviation: GV glycemic variability, HR hazard ratio, CI confidence interval, CRRT continuous renal replacement therapy, GCS Glasgow Coma Scale, APS III Acute Physiology Score III, SOFA Sequential Organ Failure Assessment

### 3.4. Subgroup analysis and sensitivity analysis

**Fig 4:**
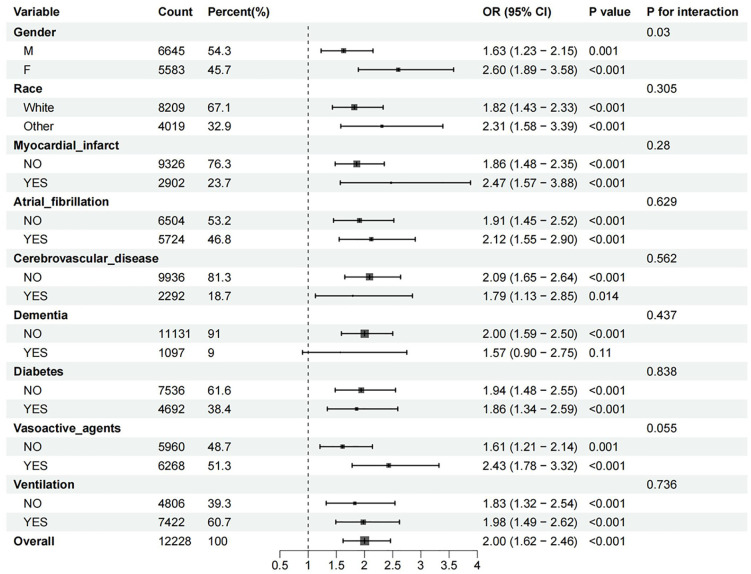
Forest plots for the association of glycemic variability with the occurrence of delirium in different subgroups.

To investigate the relationship between GV and delirium across different subgroups, analyses were performed by stratifying the study population based on sex, race (white/others), myocardial infarction, heart failure, cerebrovascular disease, dementia, diabetes, vasoactive agent, and ventilation. The findings revealed no interaction effects across all eight subgroups (Fig 4). Notably, a significant interaction was observed for gender (p for interaction = 0.03). PSM was employed to achieve a better balance among covariates across groups, and Supplementary Fig 1 illustrates the corresponding changes in matching (S2 Fig in S1 File). Within the PSM-matching cohort, the high-risk group maintained substantially prolonged durations of both ICU care and hospitalization relative to low-risk counterparts (S1 Table in S1 File). Furthermore, after PSM, CV persisted as an independent predictor of delirium among elderly sepsis patients (S2 Table in S1 File), and the association between CV and mortality risk at both 90 and 180 days remained robust (S3 Table in S1 File).

## 4. Discussion

Delirium, characterized by acute brain dysfunction, is typified by decreased cognitive ability, reduced attention span, and altered levels of consciousness [4]. SAD is a specific form of delirium occurring during sepsis, and it is notably difficult to control once it manifests [4,34]. The incidence of delirium is intricately linked to the prognosis of sepsis patients, with severe cases potentially exacerbating illness severity, prolonging hospitalization, and increasing mortality [35]. In this study, we examined the relationship between the GV cutoff of 21.39% and the incidence of delirium and mortality in elderly patients with sepsis. This cutoff is closely aligned with the median GV values across diverse ICU and sepsis cohorts [21]. Additionally, previous studies have shown that a GV level around 22% is linked to adverse neurological outcomes, further supporting the relevance of this threshold [36]. Our findings show a significant nonlinear correlation between higher GV and an increased incidence of delirium. Further analysis revealed that the high-risk group exhibited significantly higher mortality rates during both the 90-day and 180-day follow-up periods compared to the low-risk group. In addition to its role in assessing the risk of diabetes complications, these results highlight GV may serve as a clinically meaningful biomarker for identifying elderly sepsis patients at higher risk of delirium and long-term mortality.

The pathophysiological pathways linking GV to delirium in sepsis remain incompletely understood. The systemic inflammatory response triggered by sepsis has been shown to affect brain function, leading to neuroinflammation and worsening of brain dysfunction, which in turn results in delirium [14,15]. Glucose dysregulation is thought to play an important role in this process. Hyperglycemia can intensify glucose metabolic activity, contributing to elevated reactive oxygen species generation and subsequent neuroinflammatory responses [37]. Animal studies have shown that hyperglycemia is associated with barrier disruption, leading to neuronal dysfunction and incidence of delirium [38]. Severe hypoglycemia may reduce adenosine triphosphate levels, causing neuronal hyperpolarization and resulting in delirium [39]. In addition, abnormal glucose regulation can disrupt neuronal networks, contributing to the development of delirium [6,40]. Therefore, high GV may be an important risk indicator for delirium episodes in patients with sepsis.

GV is an emerging metric that quantifies fluctuations in blood glucose levels, and is a powerful tool for evaluating the relationship between metabolic status and cognitive function [10,41,42]. In young individuals, abnormal GV harms their cognition [43]. During cardiac surgery, elevated GV has been shown to increase the risk of postoperative delirium in elderly patients [40]. Moreover, several studies have established the significant prognostic value of GV in cardiovascular diseases, underscoring its importance in clinical assessment [44–47]. Nevertheless, the relationship between GV and the occurrence of delirium in sepsis patients remains unexplored. This study is the first to assess the connection between delirium occurrence and GV in ICU elderly patients, providing novel insights into this association.

Increasing evidence suggests that elderly patients with sepsis are more susceptible to the effects of GV [21]. The incidence of delirium is is known to be associated with multiple factors, including systemic inflammation, comorbidities, the use of sedatives and vasopressors, elevated blood urea nitrogen levels, reduced albumin, hypernatremia, and hypercapnia [6]. GV is influenced by hormones such as insulin and glucocorticoids [48]. However, glucocorticoid use is not significantly associated with the occurrence of delirium in postoperative patients [49]. Nevertheless, the use of insulin or glucocorticoids may still result in episodes of hyperglycemia or hypoglycemia, both contributing to the risk of delirium [14]. Benzodiazepines have been shown to be associated with the development of delirium, and for patients at risk of delirium, dexmedetomidine is generally preferred for sedation [50,51]. Furthermore, we noted that nutritional support strategies have an impact on glycemic levels. Therefore, we conducted a sensitivity analysis, which showed that glycemic variability remained associated with the occurrence of delirium. Although continuous enteral nutrition may help stabilize glucose levels, it can still be influenced by circadian rhythms [52]. Moreover, the production of lactate during sepsis may lead to glycemic fluctuations [21]. Despite significant differences in precipitating factors of delirium across patient populations, our investigation demonstrated variation in how glycemic variability influences delirium development by sex, with women over 65 being at a higher risk of delirium. Another study also indicates that women over the age of 85 in intensive care units may be at a greater risk of developing delirium [53]. However, this result analysis needs to be cautious.

GV has demonstrated utility as a prognostic indicator across a variety of conditions, including acute pancreatitis, cerebrovascular disease, cardiovascular disease, and sepsis [20,47]. In critically ill patients, corticosteroid administration and excessive enteral nutrition can aggravate glycemic fluctuations [54]. A previous study revealed that in non-diabetic sepsis patients, increased GV on the first day of ICU admission is associated with increased in-hospital mortality [21]. Moreover, the impact of high GV on mortality in sepsis patients appears to increase with disease severity [22]. A meta-analysis has shown that elevated acute GV potentially serves as a predictor for mortality risk in patients with sepsis [31]. In our study, we found that a smaller GV was associated with relatively lower mortality among elderly patients with sepsis, suggesting that a high GV in the ICU may be associated with a poor prognosis even after ICU discharge. Recent research indicated that assessments such as the SOFA score, acute physiology score, and comorbidity score can predict the prognosis of sepsis patients [55, 56]. Higher scores generally indicate a poorer prognosis. However, severity of illness scales primarily focus on physiological parameters, GV can provide a more comprehensive metabolic assessment [57], which helps medical staff improve the prognosis of sepsis patients.

Notably, this study has several limitations. First, as a retrospective observational study, this research is inevitably subject to inherent biases and unmeasured confounding factors, which may influence the results to some extent. It is crucial to emphasize that the design of this study can only for the identification of associations, not causal relationships. Second, because this analysis relied on a single-center simulated database, patient inclusion was based exclusively on ICD codes without detailed verification of individual clinical data, which may introduce selection bias. Third, the use of data-driven medians as cutoff values for binary classification could potentially lead to bias. This cutoff was derived from a single-center cohort and may limit the generalizability of the results. Fourth, the exclusion of patients with fewer than three blood glucose measurements, less than one day of ICU stay or who were not evaluated for delirium may have contributed to selection bias. These patients may represent either more severely or less severely ill cases, as suggested by their lower monitoring frequency and shorter ICU stays. Therefore, future studies should aim to validate these findings in larger, more diverse cohorts and evaluate whether interventions targeting blood glucose fluctuations can directly reduce the incidence of delirium and mortality.

## 5. Conclusion

Our study indicates that higher GV is significantly associated with an increased risk of delirium and higher mortality rates at 90 and 180 days, suggesting that continuous glucose monitoring is helpful to improve the prognostic evaluation of these individuals. In addition, this study emphasizes the importance of stable control of blood glucose levels to improve the prognosis of elderly patients with sepsis.

## Supporting information

S1 File S1 Fig. Determination of the optimal cutpoint for glycemic variability. S2 Fig. Standardized mean differences before and after propensity score matching. S1 Table. Baseline characteristics of elderly patients with sepsis in the ICU after propensity score matching. S2 Table. The association between glycemic variability and delirium after propensity score matching. S3 Table. The association between glycemic variability and mortality after propensity score matching.
(ZIP)
