# Supplementary material for: Relationship of glycemic variability with delirium and mortality among critically ill elderly patients with sepsis: A retrospective matched cohort study
Source: PLoS One. 2026 May 18;21(5):e0339707. doi: 10.1371/journal.pone.0339707 (PMC13183229; doi:10.1371/journal.pone.0339707)
Supplement: S1 File — S2 Fig. Standardized mean differences before and after propensity score matching. S1 Table. Baseline characteristics of elderly patients with sepsis in the ICU after propensity score matching. S2 Table. The association between glycemic variability and delirium after propensity score matching. S3 Table. The association between glycemic variability and mortality after propensity score matching. (ZIP) [file pone.0339707.s001.zip › supporting information/S1 Table..docx]

| **S1 Table. Baseline characteristics of elderly patients with sepsis in the ICU after propensity score matching** | | | | |
| --- | --- | --- | --- | --- |
| Variability | Overall | Low-risk group  GV＜21.39%* | High-risk group  GV≥21.39%* | P-value |
|  | (n=8558) | (n=4279) | (n=4279) |  |
| **General characteristics** | |  |  |  |
| Age, years | 77.04 (70.95, 83.91) | 77.21 (71.09, 83.68) | 76.93 (70.83, 84.16) | 0.427 |
| Race |  |  |  | 0.999 |
| White | 5816 (68.0) | 2913 (68.1) | 2903 (67.8) |  |
| Black | 843 (9.9) | 419 (9.8) | 424 (9.9) |  |
| Asian | 252 ( 2.9) | 127 ( 3.0) | 125 (2.9) |  |
| Hispanic | 207 (2.4) | 103 (2.4) | 104 (2.4) |  |
| Other | 1440 (16.8) | 717 (16.8) | 723 (16.9) |  |
| Sex |  |  |  | 0.696 |
| Male | 4607 (53.8) | 2294 (53.6) | 2313 (54.1) |  |
| Female | 3951 (46.2) | 1985 (46.4) | 1966 (45.9) |  |
| ICU LOS, days | 4.06 (2.54, 7.62) | 3.92 (2.48, 7.14) | 4.23 (2.63, 7.94) | <0.001 |
| Hospital LOS, days | 10.69 (6.74, 17.81) | 10.21 (6.45, 17.15) | 11.04 (6.97, 18.64) | <0.001 |
| **Vital signs** | |  |  |  |
| Heart rate, times/min | 83.26 (73.76, 95.45) | 83.42 (73.70, 95.75) | 83.12 (73.80, 95.24) | 0.546 |
| Respiratory rate, times/min | 19.46 (17.19, 22.29) | 19.36 (17.19, 22.23) | 19.57 (17.21, 22.34) | 0.338 |
| Spo2, % | 97.08 (95.58, 98.43) | 97.06 (95.61, 98.42) | 97.08 (95.54, 98.44) | 0.747 |
| SBP, mmHg | 112.50 (104.35, 123.45) | 112.60 (104.39, 123.51) | 112.43 (104.26, 123.40) | 0.649 |
| DBP, mmHg | 58.90 (53.02, 65.33) | 58.83 (52.96, 64.96) | 59.00 (53.13, 65.67) | 0.156 |
| Temperature | 36.82 (36.60, 37.10) | 36.84 (36.62, 37.13) | 36.80 (36.59, 37.06) | <0.001 |
| **Laboratory parameters** | |  |  |  |
| BUN, mg/dL | 29.00 (20.00, 47.00) | 29.00 (19.00, 48.00) | 30.00 (20.00, 47.00) | 0.66 |
| hemoglobin, m/uL | 9.40 (7.90, 11.00) | 9.30 (7.90, 11.00) | 9.40 (7.95, 11.00) | 0.9 |
| Platelets, K/uL | 204.00 (147.00, 279.00) | 201.00 (146.00, 273.00) | 206.00 (148.50, 284.00) | 0.017 |
| WBC, K/uL | 13.80 (9.83, 18.99) | 13.60 (9.90, 18.80) | 13.90 (9.75, 19.10) | 0.256 |
| Lactate, mmol/L | 1.70 (1.20, 2.70) | 1.70 (1.20, 2.60) | 1.70 (1.20, 2.70) | 0.161 |
| Albumin, g/dL | 3.10 (2.60, 3.50) | 3.10 (2.70, 3.60) | 3.00 (2.60, 3.50) | <0.001 |
| **Medication or treatment** | |  |  |  |
| Vasoactive agent | 4440 (51.9) | 2206 (51.6) | 2234 (52.2) | 0.559 |
| Ventilation | 5243 (61.3) | 2617 (61.2) | 2626 (61.4) | 0.859 |
| Benzodiazepines | 1826 (21.3) | 907 (21.2) | 919 (21.5) | 0.772 |
| Enteral Nutrition | 2786 (32.6) | 1409 (32.9) | 1377 (32.2) | 0.475 |
| Parenteral Nutrition | 267 (3.1) | 132 (3.1) | 135 (3.2) | 0.901 |
| CRRT | 564 (6.6) | 271 (6.3) | 293 (6.8) | 0.36 |
| Corticosteroid | 8325 (97.3) | 4164 (97.3) | 4161 (97.2) | 0.894 |
| Insulin | 7011 (81.9) | 3491 (81.6) | 3520 (82.3) | 0.432 |
| **Disease severity scores** | |  |  |  |
| GCS | 15.00 (14.00, 15.00) | 15.00 (14.00, 15.00) | 15.00 (14.00, 15.00) | 0.839 |
| SAPS II | 43.00 (36.00, 52.00) | 43.00 (36.00, 52.00) | 44.00 (37.00, 52.00) | 0.166 |
| APS III | 50.00 (39.00, 63.00) | 50.00 (39.00, 63.00) | 50.00 (40.00, 63.00) | 0.572 |
| Charlson Comorbidity Index | 6.85 (2.55) | 6.83 (2.57) | 6.86 (2.53) | 0.564 |
| SOFA | 3.00 (2.00, 4.00) | 3.00 (2.00, 4.00) | 3.00 (2.00, 4.00) | 0.466 |
| **Comorbidities or symptoms** | |  |  |  |
| Myocardial infarct | 2008 (23.5) | 1014 (23.7) | 994 (23.2) | 0.628 |
| Congestive heart failure | 3914 (45.7) | 1962 (45.9) | 1952 (45.6) | 0.845 |
| Peripheral vascular disease | 1353 (15.8) | 635 (14.8) | 718 (16.8) | 0.015 |
| Cerebrovascular disease | 1512 (17.7) | 782 (18.3) | 730 (17.1) | 0.148 |
| Dementia | 785 ( 9.2) | 393 ( 9.2) | 392 ( 9.2) | 1 |
| Chronic pulmonary disease | 2731 (31.9) | 1300 (30.4) | 1431 (33.4) | 0.003 |
| Paraplegia | 529 ( 6.2) | 286 ( 6.7) | 243 ( 5.7) | 0.059 |
| Renal disease | 2941 (34.4) | 1475 (34.5) | 1466 (34.3) | 0.856 |
| Atrial fibrillation | 4003 (46.8) | 2012 (47.0) | 1991 (46.5) | 0.665 |
| Cancer | 1523 (17.8) | 748 (17.5) | 775 (18.1) | 0.462 |
| Hypertension | 3357 (39.2) | 1681 (39.3) | 1676 (39.2) | 0.929 |
| Diabetes | 3159 (36.9) | 1564 (36.6) | 1595 (37.3) | 0.502 |
| Liver disease | 918 (10.7) | 455 (10.6) | 463 (10.8) | 0.807 |
| **Outcome** |  |  |  |  |
| Delirium | 2113 (24.7) | 1015 (23.7) | 1098 (25.7) | 0.04 |
| 90-day mortality | 2988 (34.9) | 1388 (32.4) | 1600 (37.4) | <0.001 |
| 180-day mortality | 3472 (40.6) | 1623 (37.9) | 1849 (43.2) | <0.001 |
| Data are presented as mean (SE) or frequencies (percentage).  Abbreviation: ICU LOS, icu length of stay, Hospital LOS, hospital length of stay, SBP, systolic blood pressure, DBP diastolic blood pressure, SpO2 blood oxygen saturation, BUN blood urea nitrogen, WBC white blood cell, CRRT Continuous Renal Replacement Therapy, GCS Glasgow Coma Score, SAPS II Simplified Acute Physiology Score, APS III Acute Physiology Score III, SOFA Sequential Organ Failure Assessment  *The optimal cutoff value of GV was calculated to be 21.39% to divide the population into low-risk and high-risk groups | | | | |
