# Supplementary material for: Relationship of glycemic variability with delirium and mortality among critically ill elderly patients with sepsis: A retrospective matched cohort study
Source: PLoS One. 2026 May 18;21(5):e0339707. doi: 10.1371/journal.pone.0339707 (PMC13183229; doi:10.1371/journal.pone.0339707)
Supplement: S1 File — S2 Fig. Standardized mean differences before and after propensity score matching. S1 Table. Baseline characteristics of elderly patients with sepsis in the ICU after propensity score matching. S2 Table. The association between glycemic variability and delirium after propensity score matching. S3 Table. The association between glycemic variability and mortality after propensity score matching. (ZIP) [file pone.0339707.s001.zip › supporting information/S3 Table..docx]

| **S3 Table. The association between glycemic variability and mortality after propensity score matching** | | | | | |
| --- | --- | --- | --- | --- | --- |
| Outcome | GV (continuous) | | Low-risk | High-risk | |
|  | HR (95% CI) | P |  | HR (95% CI) | P |
| 90-day mortality | |  | |  | |
| PSM Model | 1.297(1.151-1.462) | <0.001 | Reference | 1.176(1.094-1.264) | <0.001 |
| 180-day mortality | |  | |  | |
| PSM Model | 1.319(1.179-1.475) | <0.001 | Reference | 1.172(1.097-1.253) | <0.001 |
| Abbreviation: PSM propensity score matching, HR hazard ratio, CI confidence interval. | | | | | |
